# High-Resolution, Transparent, and Flexible Printing of Polydimethylsiloxane via Electrohydrodynamic Jet Printing for Conductive Electronic Device Applications

**DOI:** 10.3390/polym14204373

**Published:** 2022-10-17

**Authors:** Rizwan Ul Hassan, Shaheer Mohiuddin Khalil, Saeed Ahmed Khan, Shahzaib Ali, Joonkyeong Moon, Dae-Hyun Cho, Doyoung Byun

**Affiliations:** 1 Department of Mechanical Engineering, Sungkyunkwan University, Suwon 16419, Korea; 2 Department of Electrical Engineering, Sukkur IBA University, Sukkur 79165, Pakistan; 3 Department of Mechatronics Engineering, Gyeongsang National University, Jinju 52725, Korea; 4 Department of Energy System Engineering, Gyeongsang National University, 33 Dongjin-ro, Jinju 52828, Korea

**Keywords:** electrohydrodynamic printing, viscoelastic ink, strain sensor

## Abstract

In the field of soft electronics, high-resolution and transparent structures based on various flexible materials constructed via various printing techniques are gaining attention. With the support of electrical stress-induced conductive inks, the electrohydrodynamic (EHD) jet printing technique enables us to build high-resolution structures compared with conventional inkjet printing techniques. Here, EHD jet printing was used to fabricate a high-resolution, transparent, and flexible strain sensor using a polydimethylsiloxane (PDMS)/xylene elastomer, where repetitive and controllable high-resolution printed mesh structures were obtained. The parametric effects of voltage, flow rate, nozzle distance from the substrate, and speed were experimentally investigated to achieve a high-resolution (5 µm) printed mesh structure. Plasma treatment was performed to enhance the adhesion between the AgNWs and the elastomer structure. The plasma-treated functional structure exhibited stable and long strain-sensing cycles during stretching and bending. This simple printing technique resulted in high-resolution, transparent, flexible, and stable strain sensing. The gauge factor of the strain sensor was significantly increased, owing to the high resolution and sensitivity of the printed mesh structures, demonstrating that EHD technology can be applied to high-resolution microchannels, 3D printing, and electronic devices.

## 1. Introduction

Printing technology has developed rapidly, owing to its low cost, excellent resolution, and expedited manufacturing processes. Innovative printing techniques can create printed patterns or devices using various solution-based nanomaterials as functional inks. A detailed understanding of fluid dynamics is required because the printing process primarily entails the transfer of solution-based inks from the tip of the nozzle to the substrate. PDMS is a viscoelastic liquid that can be patterned and printed into different structures. PDMS is well suited for use in cell-based assays and cell culture and is commonly used because of its gas permeability, easy handling, lower cytotoxicity, and transparency compared with other microfabrication materials. Furthermore, it can be used in a variety of applications, such as stamps for transfer printing [1,2], lab-on-a-chip [3], nature-inspired dry adhesive layers [4,5], flexible/stretchable electronics, and microlens arrays [6,7,8]. Microelectromechanical system (MEMS) fabrication techniques, such as photolithography and electron-beam lithography, can replicate transparent and soft materials from a mold assembly. Although the entire MEMS process is expensive and time-consuming, direct printing could be an excellent way to overcome these constraints.

Additive manufacturing has recently gained popularity in education, industry, architecture, and healthcare and is useful for artificial tissue scaffolds, biological models and electronic devices made from 3D images by computed tomography or magnetic resonance imaging, and product prototyping [9]. For instance, PDMS sealants that can be vulcanized at room temperature and which have thixotropic flow characteristics have been utilized to 3D print multimaterial devices, such as reactionware for chemical synthesis [10] and bionic parts [11]. Similarly, synthetic spider webs and fluidic chambers [12,13] have been 3D printed using PDMS elastomers. The viscosity of the PDMS prepolymer can be changed by adding filler components, such as wax microparticles; this can further endow the 3D printed object with a thermos-responsive behavior once it has dried [14]. However, additive manufacturing lacks high resolution and can only be used to fabricate low-resolution devices. An alternative technique must be devised to build PDMS-based high-resolution structures such that elastomeric ink direct printing can aim to adopt a new method for printing high-resolution and flexible substrates, which is expensive using MEMS and difficult using conventional 3D printing.

Different methods for the direct assembly, patterning, and geometrical arrangement of micro- and nanoscale materials that have potential applications in chemical sensing, tissue engineering, and energy harvesting are gaining attention. In particular, electric fields provide a dependable and efficient method for utilizing local surface charges. Several unconventional jet printing methods, such as atomic force microscope charge writing [15] pyro-electrospinning [16], and bipolar electrospinning [17] have demonstrated the capacity to produce direct patterns for manufacturing high-value structures. Researchers have also worked on direct ink writing [18,19,20,21,22], laser writing [23,24,25,26], and pressure inkjet printing [27,28,29] techniques to obtain high-resolution structures of different materials. Although the aforementioned methods have been utilized to create a variety of structures, their low resolution is a major issue in increasing device functionality.

For the direct patterning of liquid materials at micro and nanoscale resolutions, EHD printing technology indeed represents a great method that aligns materials over a large area by the combined mechanism of the shear effect and mechanical stretching of the charged jet caused by the velocity distribution [30,31,32]. Instead of using acoustic or piezoelectric forces to propel droplets or jets toward a substrate, EHD jet printing uses suitable electric fields. Based on this phenomenon, micro- and nanoscale-printed electronics can be efficiently created using sacrificial or functional inks by printing a high-resolution pattern (<1 µm) on a variety of surfaces. During the EHD jet printing process, the fluid dynamics induced by the electric field also affect the geometry of the printed materials [33,34,35]. A printed web structure has been previously fabricated using EHD direct writing [36] without considering electric forces, with the structure also having a low resolution.

Based on our knowledge so far, in this study, we report for the first time the fabrication of a high-resolution mesh-printed strain sensor via the EHD route with PDMS/xylene ink. Viscoelastic ink (PDMS/xylene) was used through a microscale nozzle to study the dynamics of printing under the EHD effect. PDMS cross-linking produces time-dependent viscosity that may influence printing and curing during the patterning process. Due to the low surface energy and flowability of the viscous PDMS/xylene ink, high-resolution mesh structures were produced by varying the speed and voltage settings of the EHD printing technique. Experiments were conducted to examine the parametric effects of the voltage, flow rate, nozzle distance from the substrate, and speed to achieve a high-resolution printed structure. Furthermore, conductive silver nanowires (AgNWs) were coated by modifying their surface wettability to build functional devices, such as strain sensors. Our fabricated strain sensor showed promising resolution characteristics with the EHD jet printing technique over other printed techniques.

## 2. Materials and Methods

Preparation of PDMS ink: Dow Corning Inc. (Midland, MI, USA) provided the PDMS elastomer (Sylgard 184-A) and curing agent (Sylgard 184-B). The PDMS elastomer and curing agent were mixed by 10:1 to make PDMS ink. Xylene 0.5% wt. was mixed with the PDMS ink to make a conductive solution. Mesh printing of elastomer PDMS ink: To print PDMS ink, a multipurpose EHD printing machine (Enjet Inc., Suwon-si, Korea) was used under electric field effects. The nozzle on the Z-axis and the substrate stage on the X–Y axes can be moved by employing computer-aided controller software developed by Enjet. A microsyringe pump was used to inject 1mL of PDMS/xylene ink (Table 1) into the 34G (60 µm I.D.) plastic nozzle. The flow rate of the PDMS/xylene ink was maintained at 0.3 µL/min to ensure a constant flow. The distance between the nozzle tip and the substrate was increased from 20 to 40 µm. Mesh structures were created using trichloro (1H,1H,2H,2H-perfluorooctyl) silane (Sigma-Aldrich, Inc., Seoul, South Korea)-terminated glass substrates. To create an open microchannel array in the longitudinal direction (X-direction), the sample was soft-baked at 70 °C for 8 min. A glass substrate with an open microchannel array was then printed again in the vertical direction (Y-axis). The entire pattern of the final elastomer PDMS/xylene mesh structure was cured at 80 °C for 90 min in a convective oven.

Surface treatment of mesh-printed elastomer structure: The printed elastomer structures were first coated with an adherent polydopamine (PDA) by simply immersing them for 10–16 h in an alkaline dopamine HCl solution (3 mg of dopamine HCl dissolved in 10 × 10^−3^ M, Tris buffer, pH 8.5). The inherently hydrophobic PDMS surface was modified into a hydrophilic substrate after coating with PDA, which improved the wetting phenomena in this experiment due to the hydroxyl group in PDA. The PDA-modified printed elastomer mesh structure was also treated with an atmospheric RF plasma system (IHP-1000, APPlasma Co., Hwaseong-si, South Korea) to create a better hydrophilic surface on a pre-cleaned printed mesh structure using acetone, ethanol, and DI water. The hydrophilic surface of the PDMS printed mesh structure was dip coated with a solution of AgNWs for 20 s, dried at 60 °C for 1 min, and annealed at 150 °C for 2 h. The dip-coating process ensures an even coat on the mesh structure while the surface activation process provides sufficient adhesion.

Measurement of printed strain sensor and I–V curve: A strain sensor stretching test was performed on a custom-built testing machine by connecting the copper film to each side of the printed mesh sensor and the measurement was performed using commercially available software. Two ends of the printed mesh structure sample were attached to motorized moving stages to test their strain-sensing capabilities. Then, uniform strain/release cycles were applied and hysteresis measurements were taken at a displacement rate of 0.50 mm/s. A source meter was used to measure the relative change in resistance (Keithley 2400, Keithley Instruments Inc., Cleveland, OH, USA). We used the four-point probe measuring technique (MST 2000 A) to measure the resistivity and current–voltage (I–V) characteristics.

## 3. Results and Discussion

**Figure 1:**
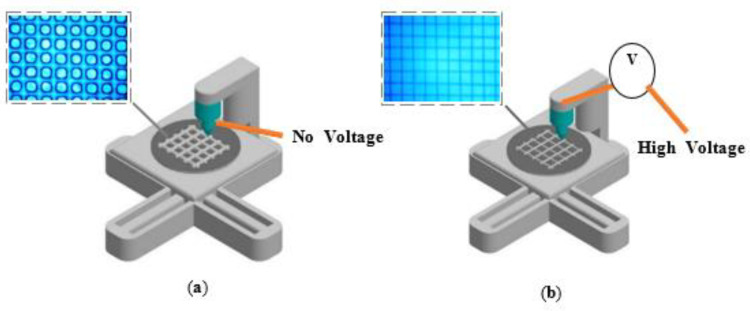
Two types of printing methods to fabricate microscale PDMS mesh patterns. (**a**) Direct ink writing and (**b**) electrohydrodynamic jet printing.

**Figure 2:**
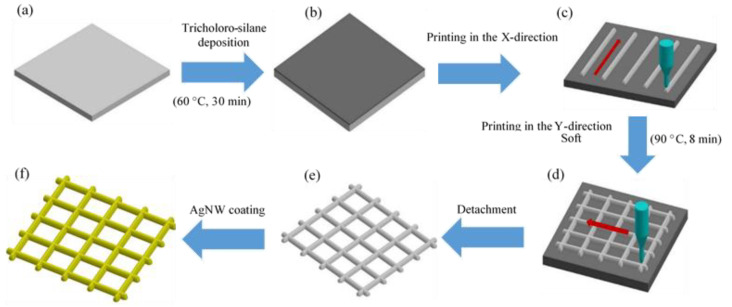
Procedure to obtain a free-standing PDMS mesh pattern. (**a**) A glass substrate, (**b**) a schematic of the trichloro-silane deposition process on a glass substrate and mesh pattern to obtain a functional mesh structure, (**c**) a printing step for the fabrication of the PDMS line in the X-direction, (**d**) a printing step for the fabrication of the PDMS mesh structure in the Y-direction, (**e**) detachment of the PDMS mesh structure from the glass substrate, and (**f**) AgNW coating process on the detached PDMS mesh structure.

Figure 1a,b illustrate the schematic of two types of printing processes to print microscale PDMS/xylene mesh patterns with direct ink writing and EHD jet printing, respectively. EHD jet printing is a non-contact printing method that induces ink ejection from a conductive nozzle onto a substrate using an electric field. Ink will not flow through a capillary nozzle, as shown in Figure S1, unless a force is applied to overcome the surface tension and capillary forces that keep it stationary. The only forces acting on it are gravity and any pressure within the system (due to the syringe pump or applied air pressure). When an electric potential is applied to the system, the charge migrates to the ink’s meniscus surface; once enough charge has accumulated on the ink interface, an electrostatic potential (normal and tangential components) is created between the meniscus and the grounded substrate. Electric forces attract the ink to the deposition substrate, where normal stress destabilizes the meniscus, while tangential stress promotes the formation of the meniscus into a cone, resulting in jetting. The normal component of the electric field is enough to overcome the ink’s surface tension. The translation stage holds the printed substrate and controls how the deposition pattern is formed, which is usually a predetermined design loaded into the control software. This stage can also move in the z direction, which modulates the strength of the electric field in the case of constant potential. A constant elastomer ink flow supplied through the microcapillary nozzle of a syringe pump was used to draw a mesh structure on a slide glass substrate using computer-aided design tools. To create a hydrophobic surface, heat deposition (60 °C, 30 min) was used for the detachment and freestanding of the final printed mesh pattern. Figure 2 shows the steps involved in printing the PDMS/xylene mesh pattern on a glass substrate. A fluorine-terminated self-assembled monolayer hydrophobic substrate was created via heat deposition at 60 °C for 30 min (Figure 2b). First, the line array was printed and soft-baked at 70 °C for 8 min (Figure 2c); then, the liquid PDMS/xylene was patterned vertically in the Y-direction (Figure 2d). To obtain a freestanding PDMS mesh pattern, it was detached from the glass substrate after hard baking at 80 °C for 90 min (Figure 2e). In our experiment, the minimum hole size was approximately 20 µm; however, this could be changed further by carefully balancing the printing parameters with the liquid properties. The printed mesh pattern should be able to operate in efficient functional devices using extra coating and printing techniques to create electronic circuits. Attaching functional materials to a hydrophobic surface without an adhesive layer is challenging because of the low surface energy of the PDMS/xylene mesh pattern and the weaker attractive molecular forces. The hydrophobic PDMS surface should be converted into a hydrophilic surface; this can enhance the wettability in this type of experiment because of the hydroxyl groups. Therefore, the printed mesh structure was plasma treated (O_2_) to render the surface hydrophilic. The AgNWs were applied to the plasma-treated mesh structure to create a functioning strain sensor device (Figure 2e).

**Figure 3:**
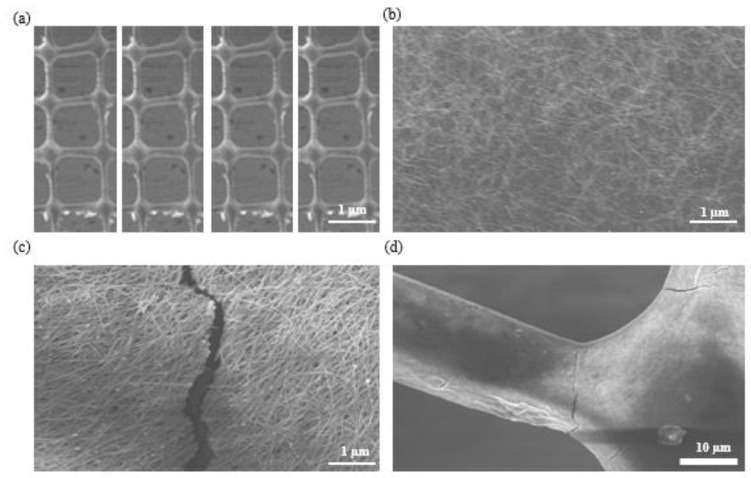
SEM analysis images. (**a**) Mesh printing structure, (**b**) uniform coating of AgNWs on the printed structure, (**c**,**d**) cracks in the coating after multiple strain tests.

**Figure 4:**
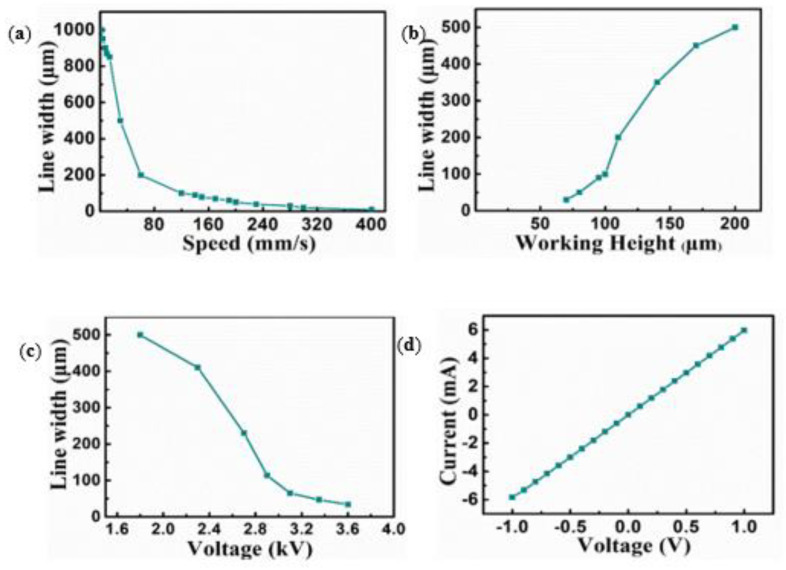
Parametric study of viscoelastic PDMS/xylene elastomer ink. (**a**) Effect of speed on the line width, (**b**) effect of nozzle height on the line width, (**c**) effect of voltage on the line width, and (**d**) I–V curve.

Figure 3 shows the scanning electron microscopy (SEM) analysis images of the AgNW coating on the printed mesh structure. The uniform coating of the printed mesh structure shows strong adherence between the AgNWs and printed structures (Figure 3a,b). Figure 3c,d show microcrack openings in the AgNWs’ thin film under stretching, indicating that the applied strain increased the electrical resistance of these thin films. Cracks are intended to form in stress-concentrated areas to release the accommodated stress. When a soft polymer is stretched, cracks form and spread in the thin films of the conducting coating on top of it [37,38,39,40,41,42]. This results in the stretching of opened and enlarged microcracks in the thin films, severely limiting the electrical conduction through the thin films due to the separation of the microcrack edges. When resistive-type sensors are stretched, the tunneling resistance can be altered. Resistive-type sensors based on AgNW-PDMS nanocomposites can possess customizable gauge factors that are controlled by the AgNW percolation network’s number density. Further, greater gauge factors and more effective separation between NW–NW connections can be achieved by lower-density networks. In this study, due to the fascinating high-resolution geometry and cracks caused by stretching, the gauge factor of the sensor was substantially high. Figure 4 illustrates the effects of the working height, voltage, and speed on the width of the printed mesh and the I–V curve results. By optimizing a speed of 350–360 mms^−1^ (Figure 4a), a working height of 50–160 μm (Figure 4b), and a voltage of 3.3–3.5 kV (Figure 4c), an optimum line width of approximately 5–10 μm was achieved. Figure 4d shows the I–V curve outcomes, presenting the relative change in resistance as a function of the given tensile strain by the stretch and release procedures that exhibit the adhesiveness of the functional AgNWs to the printed mesh structure. According to the mesh geometry, the hysteresis performance of the strain sensor can be recovered, and varied strain circumstances can be employed to efficiently control the gauge factor. The development of cracks causes a change in resistance during stretching and, hence, also affects the gauge factor.

**Figure 5:**
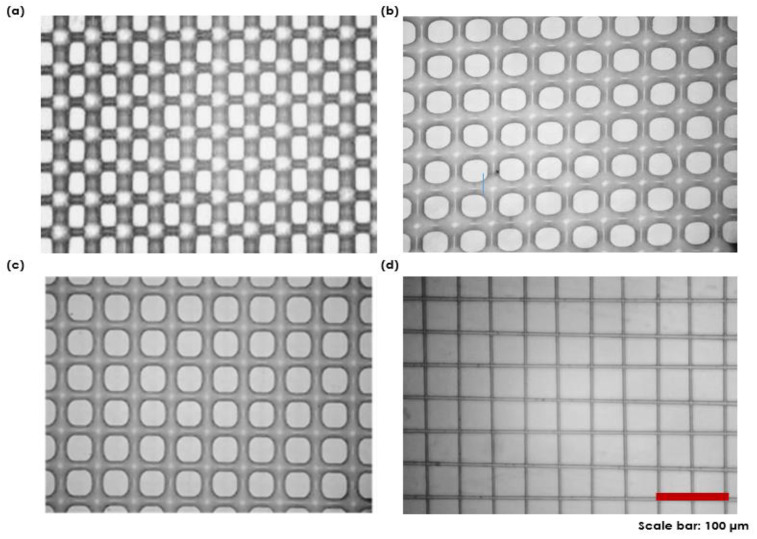
Microscopic images of PDMS mesh patterns fabricated by (**a**) direct ink writing (0 V) and (**b**) EHD jet printing at (**b**) 1.5 kV, (**c**) 2.3 kV, and (**d**) 2.8 kV.

Figure 5 shows how a mesh-printed elastomer structure design can be successfully created using direct ink writing and EHD jet printing techniques. The printed mesh structure resolution was low, at approximately 50 µm, and was achieved by direct ink writing, where no electric field effects were applied between the nozzle and substrate (Figure 5a). When we printed liquid PDMS/xylene in the perpendicular direction on the soft-baked transverse pre-patterned elastomer, the liquid PDMS/xylene formed a low-resolution round mesh structure (Figure 5a). The minimum hole size in the direct ink writing experiment was approximately 30 µm and the line thickness was around 50 µm, but it was possible to change it further by carefully altering the printing parameters (voltage, speed, and working height) along with the liquid properties. To achieve a high resolution, we also created PDMS-based mesh structures using the liquid instabilities caused by the EHD effects (Figure 5b–d). Owing to the externally applied electric field, the resolution of the mesh-printed structure was approximately 10 µm; this is approximately 10 times higher than that of the conventional direct-writing method. The hole size of the printed mesh structure was approximately 50 µm as the PDMS line thickness was shortened by electric field effects. An EHD field capable of boosting the prominent instability in the natural spectra of the capillary surface waves was applied to the air–liquid interface at an ambient temperature. The liquid PDMS/xylene might experience an EHD force from the electric field, forming a liquid bridge between the two substrates. The liquid PDMS had a height of approximately 110 µm, by four consecutive layers in the z-direction with a wetting contact angle of approximately 60° (C.A.) on a hydrophobic silane-terminated surface. When we attempted to print on bare hydrophilic glass, the aspect ratio was significantly lower than that in the hydrophobic scenario, and we were unable to create the appropriate geometry to fully analyze the printing mechanism.

**Figure 6:**
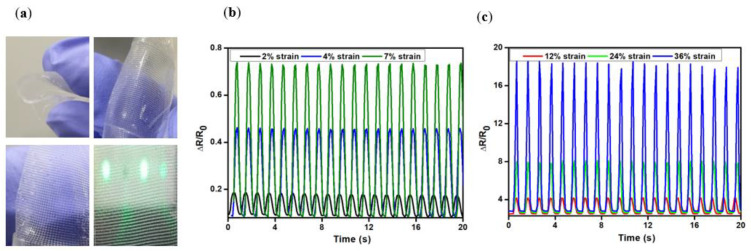
Printed mesh structure and gauge factor measurement. (**a**) Printed mesh structure flexibility and bending tests. The relative change in resistance as a function of the given tensile strain by the stretch and release processes during small (**b**) (2–7%) and large (**c**) (12–36%) strains.

An examination of the manner in which the applied tensile strain during the stretch and release procedures affected the relative resistance change ([R − R0]/R0) is shown in Figure 6. Under severe strain, resistive-type strain sensors frequently display substantial nonlinearity and hysteresis. However, in this study, at low and high strains, the hysteresis performance was almost completely recovered by the mesh structure of the printed electrodes. Additionally, a variety of strain conditions could be used to effectively adjust the gauge factor (Figure S2). The creation of cracks caused a change in the resistance during stretching (Figure 3c,d). The resistance might also be altered because of the various distances between the cracks. Several research teams have created various types of architectures (such as serpentine-shaped conducting lines or stretched substrates) to lower the maximum strain value during mechanical deformation to manufacture stretchable strain sensors. The specifications of stretchy directions and difficult fabrication procedures have limitations, although printing could be a different approach to creating stable and stretchy sensing applications to overcome these challenges. Owing to the mesh structure, a considerably higher gauge factor sensor value was achieved than those of the previous results (Figure 6), indicating that this can be used for various potential applications. A high-resolution printing technique was used to fabricate sensitive and flexible strain sensors. This technique can be further applied to high-resolution 3D printing and microchannels. We obtained a high-resolution (10 µm) mesh-printed strain sensor via the EHD jet printing technique that showed promising resolution characteristics over other printed techniques (Table S1) [36,43,44,45,46,47].

## 4. Conclusions

In this study, we proposed a new method for fabricating high-resolution stretchable mesh strain sensors using the EHD jet printing technique with PDMS/xylene elastomeric ink. We investigated the printing dynamics of high-resolution mesh structures using a microscale nozzle under EHD effects. It was found that a scalable sensor design can be fabricated by fine-tuning the rheological properties of the ink and controlling the print path and electric field effects. Experiments were conducted to investigate the parametric effects of voltage, flow rate, nozzle distance from the substrate, and speed to achieve high-resolution (5–10 µm) printed structures. In addition, we reduced and strongly immobilized the conductive AgNWs on printed mesh structures to capitalize on functional architectures, such as strain sensors. Our fabricated strain sensor showed promising resolution characteristics with the EHD jet printing technique over other printed techniques. This method can also be used to create soft functional devices for wearable electronics, human–machine interfaces, soft robotics, and other potential applications.

## Figures and Tables

**Table 1:** Ink preparation.

Materials	Weight (%)
PDMS (base)	10
PDMS (reagent) Xylene	1 0.5

## Data Availability

The data presented in this study are available on request from the corresponding author.

## Supplementary Materials

The following supporting information can be downloaded at: https://www.mdpi.com/article/10.3390/polym14204373/s1, Figure S1: Physics of EHD jet printing where forces acting on a capillary tip during EHD printing by cone jet mode; Figure S2: Various gauge factor values according to the strain; Table S1: Comparison of printing techniques and resolutions.
