# Supplementary material for: High-Resolution, Transparent, and Flexible Printing of Polydimethylsiloxane via Electrohydrodynamic Jet Printing for Conductive Electronic Device Applications
Source: Polymers (Basel). 2022 Oct 17;14(20):4373. doi: 10.3390/polym14204373 (PMC9607091; doi:10.3390/polym14204373)
Supplement: Supplementary file 1 [file polymers-14-04373-s001.zip › polymers-1960111-supplementary.pdf]

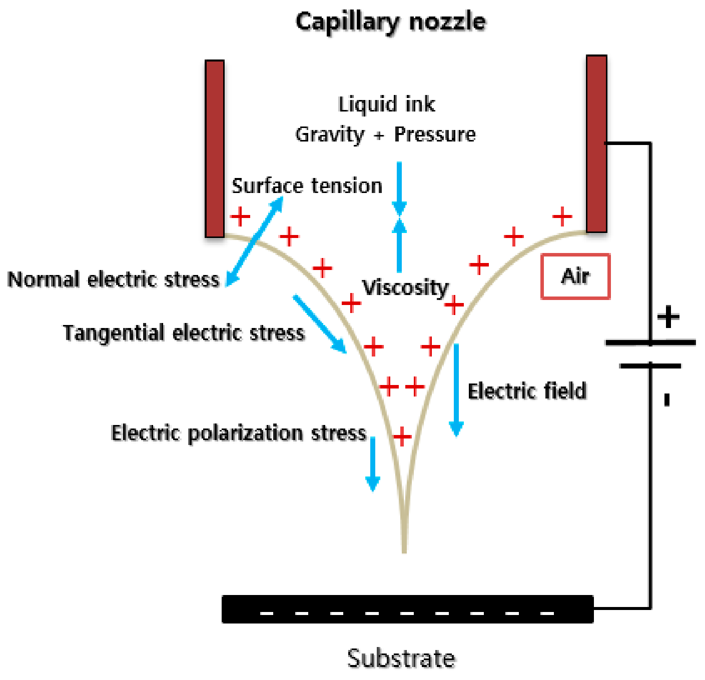

Figure S1. Physics of EHD jet printing where forces acting on a capillary tip during EHD printing by cone jet mode

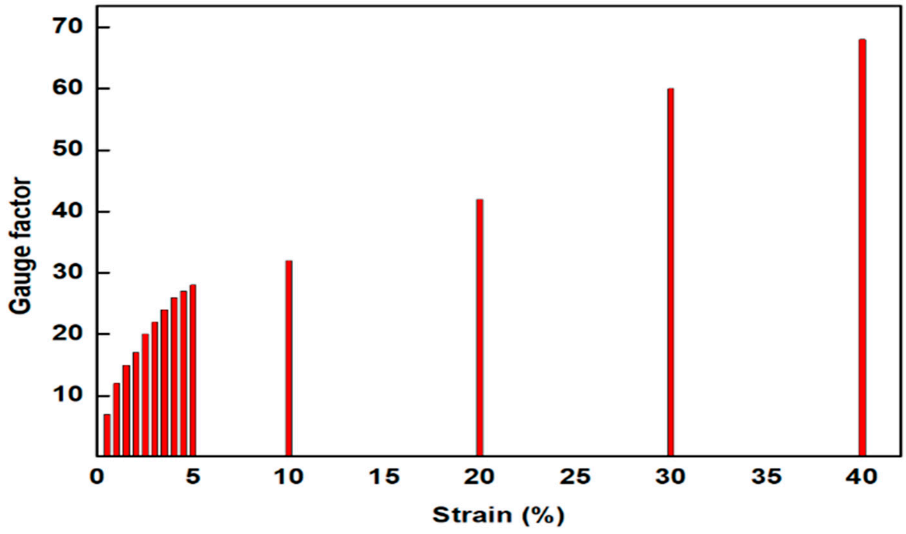

Figure S2. Various gauge factor values according to the strain

Table S1. Comparison of printing techniques and resolutions.

No.	Printing Technique	Width (μm)	Applications	Ref.
1	Direct Writing	50	Strain sensor	36
2	UV laser writing	30	Additive manufacturing	43
3	Razor writing	500	Microchannels	44
4	3D printing	150	Terahertz	45
5	3D printing	8000	Strain sensor	46
6	Mold Technique	18000	Flow sensor	47
7	This Work	5-10	Strain sensor	2022
